# Expression of cellular senescence and neural stem cell markers in a case of advanced retinal fibrosis

**DOI:** 10.1002/agm2.12094

**Published:** 2019-12-25

**Authors:** Manuel Moreno‐Valladares, Ander Matheu

**Affiliations:** ^1^ Donostia Universitary Hospital San Sebastian Spain; ^2^ Cellular oncology group Biodonostia Institute San Sebastian Spain; ^3^ Ikerbasque Basque Foundation Bilbao Spain; ^4^ CIBERfes Basque Foundation Madrid Spain

**Keywords:** AMD, cellular senescence, fibrosis, stem cells

## 1 INTRODUCTION

Fibrosis in the posterior segment of the eye tends to be the final stage in the resolution of lesions in the retina and choroid. Two of the diseases that most often cause this type of fibrosis are diabetes and age‐related macular degeneration (AMD).^1^ The main aetiopathogenetic mechanisms involved in the development of fibrosis are hypoxia and inflammation. In the case of AMD, retinal pigment epithelium dysfunction leads to accumulations of substances (drusen) with angiogenic activity on Bruch's membrane, this becoming thicker and limiting oxygen diffusion to the most external layers of the retina. It causes two types of lesions: dry or atrophic lesions, and wet or exudative (angiogenic) lesions.^1^, ^2^ On the other hand, diabetes leads to hypoxic lesions due to microvasculopathy associated with glycosylation of the vascular wall. Both processes can lead to blindness.

In AMD, cellular senescence and stem cells may play a key role in the progression of the lesions. The progressive accumulation of cell damage over time promotes the induction of senescence in which cells block their capacity to divide and acquire functions for biosynthesis and secretion of various different cytokines (senescence‐associated secretory phenotype), which in turn leads to immunological and remodelling responses of the extracellular matrix, associated with repair mechanisms in damaged tissues.^1^, ^2^, ^3^ The inflammatory damage may, however, remain and increase, despite a reparatory inflammatory response. With age there is stem cell deficiency and hence an inability to regenerate the damaged structures.^4^ The progressive accumulation of senescent cells and stem cell depletion with aging may be the basis of the deterioration of the mechanisms of tissue homeostasis activated in response to damage and, consequently, responsible for fibrosis accumulation.

## 2 CASE PRESENTATION

A 76‐year‐old man with a history of atrial fibrillation and age‐related macular degeneration treated with intravitreal injection of angiogenesis inhibitors was diagnosed with disseminated colorectal adenocarcinoma. During the course of the disease, he attended the emergency department due to an episode of acute pain, with loss of vision in his left eye. The eye examination revealed a narrow anterior chamber angle with irido‐trabecular apposition and an intraocular pressure of 34 mm Hg. A diagnosis was made of acute glaucoma and an MRI was requested, this revealing retinal detachment with subretinal haemorrhage and a nodular image in the temporal field that suggested that the differential diagnosis should include haemorrhagic metastasis, choroidal melanoma and blood clot.

The patient finally died and a post mortem examination of the left eye was performed.

**Figure 1:**
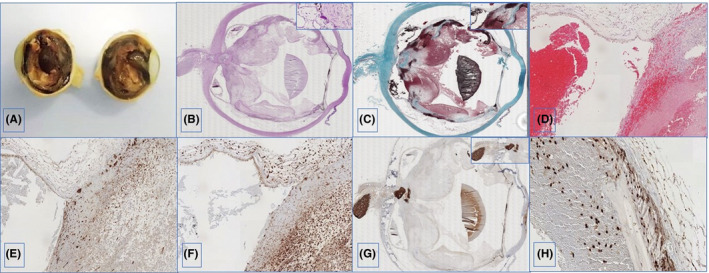
Representative images of hemorrhagic peripheral areas and central white tan fibrous (A). Small round amount of amorphous hyaline material is seen beneath retinal pigmented epithelium, called druse, which stain with PAS (detailed in box) (B). Two curvilinear horn‐shaped fibrous tissue (green) are seen at the periphery over the retinal layer (detailed in box), with abundant loose connective tissue in between (C). The retinal pigmented epithelium is conserved attached to the choroid, while the rest is substituted by abundant loose connective tissue, rich in irregular blood fine vessels, and hemorrhage. H/E stain (D). Extensive p16^Ink4a^ positive cells are seen in connective tissue and vascular structures (detailed in box) (E). Inflammatory infiltrate with abundant macrophages (F). Gial scar at optic papillae (detailed in box) (G). Isolated foci of retina partially conserved at periphery expressing SOX‐2 (H).

The eye was enucleated and fixed in 4% formaldehyde. Then a first cut was made sagittally from the anterior pole to the optic nerve, revealing a heterogeneous lesion that occupied the entire volume of the vitreous body, composed of tissue with a devitalised fibrous appearance and haemorrhagic content (Figure 1A). Subsequently, several cuts were made parallel to the first; the tissue was embedded in paraffin and histological sections were taken with a microtome. These sections were stained histochemically (haematoxylin‐eosin, PAS and Masson's trichrome stain) and immunohistochemically for analysis of stem cells (Sox‐2), cellular senescence (p16), inflammatory cells (CD‐68), vascular endothelium (CD‐31) and glial cells (GFAP).

The choroid, below the sclera, did not show any significant lesions except near the optic papilla or blind spot, where some signs of fibrosis were observed. On the other hand, the histological structure of the retina had mostly disappeared except at certain points on the periphery (ora serrata), where there were still some cells. Only the retinal pigment epithelium was almost fully intact. Between this structure and Bruch's membrane, staining (PAS positive) revealed abundant drusen (Figure 1B). There was even dense collagenous connective tissue extending along the curvature of the retina seeming to take its place (Figure 1C). In several places, the retina had detached from the choroid and adhered through the pigment epithelium to loose connective tissue with abundant capillary vascularisation and extensive haemorrhage (Figure 1D). In these tissues, there was notable expression of the marker of cellular senescence p16^INK4^ (Figure 1E), both in the fibroblastic and the endothelial components of the neoformed capillaries. Along with these lesions, there was an inflammatory exudate which mainly contained macrophages, many of which were loaded with haemosiderin (Figure 1F).

At the level of the optic papilla, we found a glial scar (Figure 1G) in highly vascularised connective tissue. Its cells expressed a marker of neural stem cells, Sox‐2, which was also observed in the peripheral retinal remanent. This was not observed, however, in the rest of the tissue with fibrosis and haemorrhage (Figure 1H).

## 3 DISCUSSION

The most significant lesions observed in this case report may be considered to be: (a) the accumulated drusen on Bruch's membrane, as a morphological expression of cell dysfunction in the pigment epithelium; (b) the extensive loose and dense connective tissue, adhering to the aforementioned epithelium in the absence of the retina; (c) the enriched vascularization; (d) the percentage of senescent cells in both the connective tissue and the tissue forming the capillaries; the macrophage‐predominant inflammatory response, laden with hemosiderin; and (e) the lack of stem cell marker expression (Sox‐2), which was practically only seen in the glial scar and the peripheral area, where some of these cells appear in retinal remnants while others appear isolated in haemorrhagic lesions. Taken together, these features may be understood as the consequences of a loss of balance between cellular senescence and stem cells, favoured by aging, as the pathophysiological expression of the mechanism for the development and progression of lesions described in the case report.

In particular, cellular senescence is involved in the vicious pathogenic circle maintaining of inflammatory activity and fibrosis but also forming abnormal blood vessels which may contribute to extensive and regular haemorrhages. On the other hand, the destruction of the retina implies the disappearance of Sox‐2 positive stem cells that appear, in isolation, in a remnant of retinal structure located in the ora serrata. Some of the positivity for Sox‐2 at this site corresponds to cells segregated from the tissue, located in the haemorrhagic lesions. This deficiency may be contributing to the damage, failure to regenerate tissue and accumulation of fibrosis. Senescence accumulation and deficiency in stem cell activity may be contributing to the damage and fibrosis accumulation in AMD.

## CONFLICTS OF INTEREST

The authors declare no conflicts of interest.

## AUTHOR CONTRIBUTIONS

*Study design, experiments and drafting of the manuscript*: Moreno‐Valladares. *Interpretation of results and substantive revision*: Matheu. Both authors have read and approved the manuscript.

## ETHICS APPROVAL AND CONSENT TO PARTICIPATE

The brother of the patient signed informed consent form approved by the Institutional Ethical Committee. The study was approved by the ethics committee of Hospital Donostia.
